# Adolescents and young adults dating and HIV perceptions: A phenomenological study in N’Djamena, Chad

**DOI:** 10.1080/17441692.2025.2534619

**Published:** 2025-07-21

**Authors:** Esias Bedingar, Ngarossorang Bedingar, Christopher Sudfeld

**Affiliations:** a Department of Global Health and Population, Harvard T.H. Chan School of Public Health, Boston, MA, USA; b Alma, Centre de Recherche en Systèmes de Santé, N’Djamena, Chad; c Croix Bleue Tchadienne, N’Djamena, Chad

**Keywords:** Adolescent dating, HIV knowledge and risk perception, peer education, sexual health behaviour, Chad

## Abstract

The study investigates the interplay between adolescent dating behaviours and HIV perceptions in Chad, where adolescents and young adults (AYA) aged 15–24 years are disproportionately affected. Four focus group discussions (n = 48) were conducted with high school students in N’Djamena, stratified into beneficiaries and non-beneficiaries of a peer education programme. Using a phenomenological approach and the Colaizzi method in ATLAS.ti, we examined lived experiences around relationships, risk behaviours, and prevention strategies. Distinct gender differences emerged in dating motivations: males prioritised sexual satisfaction, while females sought emotional connection. Partner selection was influenced by intrinsic factors (emotional attraction) and extrinsic factors (financial benefits). Awareness of PrEP was absent, and condom use was more often linked to pregnancy prevention than HIV/STI protection. Risk behaviours such as alcohol use and multiple partnerships were common, with gender roles influencing safe sex decision-making. Shared responsibility for protection was noted only among beneficiaries of the peer programme. The findings highlight the need for gender-sensitive HIV prevention interventions addressing AYA’s realities. Increased awareness of PrEP and moving beyond abstinence-focused strategies are essential to bridge gaps in HIV prevention and education in Chad.

## 1. Introduction

Young people ages 15–24 years contribute to almost half of all new HIV infections globally (World Health Organization & UNAIDS, 2011). Approximately, 80% of HIV-positive adolescents and young adults (AYA) reside in sub-Saharan Africa (SSA) (Kharsany & Karim, 2016). In Chad, the overall prevalence was 1.0% in 2022, but AYA bear a disproportionate burden (The DHS Program, 2014–2015; UNICEF, 2019). Among AYA aged 15–24 years, HIV prevalence is estimated at 1.3% for females and 0.8% for males (UNICEF, 2019), increasing notably with age among young women – from 1.2% at ages 15–19 years to 2.4% at ages 23–24 years (UNICEF, 2019). According to UNAIDS, 26.3% of new HIV infections in 2022 were among AYA, compared to 28.4% and 30.8% in 2010 and 2015, respectively (République du Tchad, Conseil National de Lutte Contre le SIDA, 2021). Despite this decline, young women continue to suffer disproportionately from the HIV epidemic, more specifically due to the ‘HIV triple threat’, which refers to the combination of new HIV infections, sexual and gender-based violence, and adolescent pregnancies (Global Communities, 2022). Young women now account for 19.5% of all new HIV infections globally, with infection rates four times as high as those of young men (République du Tchad, Conseil National de Lutte Contre le SIDA, 2021).

National interventions have focused largely on preventing mother-to-child transmission (PMTCT), with limited investment in prevention for sexually active AYA (UNICEF, 2019). This gap may be attributed to sociocultural taboos surrounding AYA sexuality, which hinder open discussions and education around reproductive health (The New Humanitarian, 2008).

Understanding the context of adolescent dating and sexual behaviour in urban centres like N’Djamena is important to address HIV risk among AYA. While early marriage remains common in rural Chad – 61% of girls are married by age 18 – urban areas like N’Djamena follow different social dynamics (Girls Not Brides, n.d.). In the capital, young women tend to marry later, creating more opportunity romantic relationships before marriage (Girls Not Brides, n.d.). Premarital sexual activity is more prevalent among young men than women, due in part to the earlier age at marriage for young women (The DHS Program, 2014–2015). Nationally, 66% of young women have had sex by age 18, but most of this is within marriage, while only 8% of young men are married by that age (DAI, 2021; Girls Not Brides, n.d.; Varelis et al., 2024). Yet, 37% of young men report sexual activity before 18, indicating that premarital sex is more common among young men (DAI, 2021). The median age at first sexual intercourse for young women is about 16, closely tied to the age of marriage, while young men typically initiate sex later, in late adolescence, and mostly outside of marriage (The DHS Program, 2014–2015; DAI, 2021). Among those who had sex before the age of 18, 21.1% of young women were single compared to 31.8% of young men, while 76.8% of young women were in a union, compared to 53.5% of young men (The DHS Program, 2014–2015).

In urban areas, these trends are magnified: later marriage, greater exposure to media, and increased social interaction between genders contribute to more dating and sexual experimentation (Omar, n.d.). Among these youth, condom use remains low – only 41.3% of sexually active young women (aged 15–24) reported using condoms during their last sexual encounter against 49.7% for young men – raising the stakes for HIV and STI transmission (The DHS Program, 2014). Additionally, alcohol use is a growing concern. Roughly one-third of Chadian youth report using alcohol or drugs, with rates likely higher in N’Djamena (Radio Ndarason Internationale, 2022). Drinking is more prevalent among young men, often occurring in informal social settings and sometimes preceding sexual activity. Some young women themselves note that alcohol increases their vulnerability to unwanted or unprotected sex (Alwihda Info, 2025; Radio Ndarason Internationale, 2022).

In this context, peer education in schools has emerged as a potential strategy for behaviour change (Faust & Yaya, 2018). Peer-led HIV education programmes in schools aim to bridge this gap by providing accurate information and fostering supportive environments and these programmes can leverage the unique position of peers who can communicate complex health messages in a relatable and understandable manner (World Health Organization, 1997). In 2012, the ‘Life Skills and Peer Education’ initiative, also known as the BCC Lifeskills Project (2012–2016), was initiated by Blue Cross Chad for at-risk students aged 14–18 in schools (International Blue Cross, 2016; International Blue Cross, 2021). This project’s goal was to enhance understanding and skills for making educated choices regarding substance abuse, alcohol, and the risks linked with HIV. The programme included peer educators trained in life skills who covered various subjects, from HIV prevention to transmission, using interactive teaching methods like group activities and role-playing.

From 2012 to 2016, the BCC Life Skills-Based Education (LSBE) was conducted in 15 high schools in N’Djamena and later expanded to 20 schools by 2021. With the desire to inform programmatic strategies for scale-up, we conducted a qualitative study to explore the experiences of AYA in reproductive health and HIV and AIDS. The study also aimed to understand how the dating culture and practices influence AYA’s knowledge, misconceptions, and prevention strategies regarding HIV. Insights from this study are intended to inform the development of effective HIV prevention and education programmes tailored to the unique context of Chadian AYA.

## 2. Methods

### 2.1. Study design

This qualitative study employed a descriptive phenomenological approach, grounded in the philosophical principles of Edmund Husserl (Cresswell, 1994). This methodology is particularly effective in capturing and understanding the unique perspectives and experiences of the participants, especially when exploring phenomena like HIV-supportive norms among AYA (Brinkmann & Kvale, 2009; Jackson et al., 2018). Husserl’s four-step process of descriptive phenomenology – bracketing, intuiting, analyzing, and interpreting – guided the research (Husserl, 1964). To maintain objectivity, the research team engaged in regular discussions to set aside preconceived notions, a process known as bracketing, ensuring that the participants’ lived experiences remained central to the analysis (Husserl, 1964).

### 2.2. Study setting

The study was conducted in N’Djamena, the capital and largest city of Chad, with a population of approximately 1 538 387 people. The city comprises 10 districts and was purposefully selected due to the implementation of the Blue Cross Chad (BCC) LSBE programme in 20 local high schools (Wikipedia contributors, 2023). As of 2022, the HIV prevalence in Chad stood at 1.0%, with AYA disproportionately affected by the epidemic (The DHS Program, 2014).

### 2.3. Participants and sampling

Participants were selected using purposive criterion sampling to include students from both schools participating in the BCC LBSE programme and those not involved in the programme, facilitating a comparative evaluation of the programme’s impact. The study formed four focus group discussions (FGDs), each comprising 12 participants, totalling 48 students. The groups were stratified by gender, resulting in two groups of young women and two groups of young men. Inclusion criteria encompassed students currently enrolled in grades 9–12. For those in participating schools, a minimum of six months’ involvement in the programme was required to ensure adequate exposure to the intervention.

The sample size was determined based on the study’s exploratory nature and logistical considerations. While empirical saturation was not achieved, the selected sample provided diverse perspectives essential for the study’s objectives. The decision to include 12 participants per group, exceeding the typical FGD size of 6–8, aimed to enhance the diversity of viewpoints and enrich group dynamics.

### 2.4. Data collection

Data collection occurred between October 4th and 6th, 2022. Six trained interviewers, organised into two gender-specific teams, with two female interviewers facilitating all FGDs with female AYA, and two male interviewers facilitating all FGDs with male AYA. While each FGD was facilitated by a team of three interviewers, the inclusion of female interviewers ensured cultural sensitivity and participant comfort, particularly in discussions involving female AYA.

FGDs were carried out in French and Arabic using a semi-structured guideline (S1 Appendix). Sessions took place in a private and secure location at the BCC headquarters, each lasting between 90 to 120 min. All discussions were audio-recorded, transcribed verbatim, and translated into English collaboratively by the research team to preserve cultural nuances and contextual meanings.

### 2.5. Data analysis

Data analysis was conducted using ATLAS.ti version 22, employing Colaizzi’s seven-step method, which emphasises participants’ perspectives to present an authentic understanding of their experiences (Colaizzi, 1978; Lincoln & Guba, 1985; Scientific Software Development GmbH, 2016). The steps included familiarisation, identifying significant statements, formulating meanings, clustering themes, developing an exhaustive description, producing the fundamental structure, and seeking verification of the fundamental structure (Colaizzi, 1978). The analysis was a collaborative effort involving all team members, including Chadian researchers, ensuring cultural relevance and accuracy. Themes and sub-themes were identified through consensus, and the coding tree’s dependability was affirmed through team discussions on code definitions and inclusion criteria.

### 2.6. Trustworthiness

To ensure the integrity and consistency of the data, known as trustworthiness, the team applied Lincoln and Goba’s evaluative criteria, encompassing credibility, transferability, dependability, and confirmability (Jackson et al., 2018). The researchers extracted and categorised text associated with the codes, comparing similarities and differences. Given that the interviewers were locals with an understanding of the regional culture, they actively participated in this analytical process. The coding tree’s dependability was affirmed through a consensus among team members on the definitions and criteria for code inclusion or exclusion. Confirmability was attained by meticulously documenting and archiving all stages of the research process, including multiple revisions and discussions of the primary and secondary categories identified by the researchers.

### 2.7. Reflexivity

In terms of reflexivity, the diverse and multinational qualitative research team, proficient in multiple languages and disciplines, worked to minimise potential biases in the analysis. The inclusion of locally trained interviewers aided in capturing cultural nuances and facilitated acceptance within the community. This aspect of the research was reflexive and collaborative, engaging continuously with the NGO partner, school and government officials, and development organisations. Despite the team’s diverse composition, potential biases in the conclusions could not be entirely ruled out. To address this, triangulation was applied, involving sharing findings with community members not interviewed and experts on the subject to identify possible inaccuracies or biases overlooked by the research team.

## 3. Results

Data collection included forty-eight in-classroom high school students divided into four FGDs. Each group was gender-specific, with two groups composed of female students and two of male students, to ensure participant comfort given the sensitive nature of the topics and prevailing gender norms in Chad. This stratification aimed to encourage open and honest discussion, particularly among female participants who may have felt less comfortable speaking in mixed-gender settings. Groups were not further stratified by age or grade level; students from grades 9 to 12 were included in each group. Of the 48 participants, the majority were enrolled in 10th (n = 17) and 11^th^ (n = 18) grades, representing 73% of the sample (Table 1). The mean age of participants was 16.95 years (SD = 0.90), with slight variations by subgroup. The distribution of participants included 24 students (12 male and 12 female) from schools participating in the BCC programme and 24 (12 male and 12 female) from non-participating schools (Bedingar et al., 2024). Participant IDs were anonymized and known only to the research team.

Guided by descriptive phenomenology, the analysis of the AYA’s lived experiences revealed two overarching themes: the culture of dating and risk-taking behaviours. Each theme comprised several sub-themes, as outlined in Table 2. The following sections present these findings in detail, incorporating illustrative quotes from participants to highlight key insights related to dating norms and HIV prevention among AYA.

### 3.1. Taking a step back: What is the culture of dating among the AYA in Chad?

#### 3.1.1. Reasons for dating

Students expressed various reasons for dating; however, two main categories emerged, including those who were in a relationship because they were seeking companionship and others whose motivations were driven by sexual behaviour. Among participants, dating was associated with emotional closeness and meaningful connection. For example, one young man described dating as ‘*a friend to share ideas with’* (PM, #4, 19-years), emphasising intellectual and emotional partnership. Among young women, the search for companionship was shared as the primary reason for dating. Several participants framed dating as a way to envision the future together, stating that ‘*what is involved in dating is to have a healthy relationship, getting married, and having a family’* (PF, #14, 16-years). Another noted, ‘*we go out with someone to change ideas about the future and talk about behaviors to change’* (NPM, #35, 16-years), reinforcing the idea of shared planning and moral development in dating relationships. However, these emotionally anchored motivations were contrasted by other participants who framed dating in more casual or transactional terms. Statements like ‘*we go out with someone to share gifts and take selfies’* (NPM, #27, 19-years) and ‘*you date someone to have memories and to give them what they want’* (NPM, #27, 19-years), reflect performative or material motivations, particularly among AYA in non-participating schools.

Some young women also reported that initial emotional motivations could evolve due to social or partner pressure. As one young woman from a participating school shared, ‘*I believe what motivates young people like me to practice sex is peer pressure and desire’* (PF, #20, 15-years). On the other hand, sexual motivation was more frequently expressed among young men. One young man framed sex within dating as a marker of masculinity: ‘*This means that for us, sex is a sign of maturity, or it means that we want to test our masculinity’* (PM, #11, 16-years), while another plainly stated that ‘*men of [their] age have sex just to satisfy themselves and for pleasure’* (NPM, #28, 17-years). Others shared, ‘*as men, I believe that we go out with girls because we admire them, but also to chat and have sex’* (NPM, #26, 16-years). These quotes illustrate that dating can also function as a space where masculinity identity and sexual expectations are affirmed. Overall, these diverse responses underscore how dating is understood not only as a pathway to intimacy and future partnership but also as a space influenced by peer dynamics, desire, and gender norms.

#### 3.1.2. Partner selection criteria

Participants highlighted both intrinsic and extrinsic motivations in choosing a partner (Table 3). Emotional and family-oriented considerations were common. As one young woman explained, ‘*the culture around dating is focused on emotional attraction and sharing ideas’* (PF, #21, 18-years), while a young man mentioned, ‘*what is involved in dating is to … end up with a marriage’* (PM, #5, 16-years), underscore the personal and value-based dimensions of partner choice. On the other hand, extrinsic factors such as physical appearance and material benefits were also frequently cited. One young man noted, ‘*Our choice of partners is based on physical appearance’* (PM, #3, 17-years), while a young woman from a non-participating high school stated: ‘*We date men because of money’* (NPF, #39, 17-years). A male counterpart admitted that for him, ‘*[He] chooses [his] partner based on the fact that she is helpful, and she offers [him] gifts’* (NPM, #26, 16-years). These quotes underscore how material expectations influence dating preferences, particularly among AYA in non-participating schools.

### 3.2. Awareness and risk-taking behaviours about HIV

The previous section dissected the nuances of dating culture among AYA in Chad, analyzing their motivations, behaviours, and the social contexts of their romantic relationships. The following section presents a detailed examination of AYA’s understanding and management of HIV risks, elucidating the critical connection between their dating lives and vulnerability to HIV.

#### 3.2.1. HIV knowledge

Participants who had received peer education through the BCC programme frequently cited changes in their knowledge about HIV. One young woman stated, ‘*Our knowledge [of HIV] has changed through Blue Cross Chad’* (PF, #16, 16-years), and another young man added that the programme ‘*shaped [our] beliefs about HIV and AIDS’* (PM, #8, 19-years). This enhanced understanding appeared to influence behaviour, as illustrated by a student who explained, ‘*[We] could now practice it responsibly’*(PM, #10, 18-years), suggesting greater awareness and preparedness in managing sexual health. For example, a beneficiary correctly explained that HIV ‘*could be transmitted from a mother to a child during childbirth, or through unprotected sex with an infected person, or soiled objects’* (PF, #18, 18-years). In contrast, several misconceptions were observed among students from non-participating schools. One young man incorrectly stated, ‘*[It is] a contagious disease that is transmitted through blood and mosquitoes’* (NPM, #32, 16-years), while another cited ‘*skin contact’* as a possible transmission route (NPM, #35, 16-years).

#### 3.2.2. HIV risk-taking behaviours

Multiple risk behaviours were identified, particularly alcohol use and having multiple sexual partners. A young woman commented: ‘*I believe that women of my age takes risks when they are drunk or intoxicated’* (NPF, #41, 19-years). Another added, ‘*risks come from having multiple partners or even ignorance’* (NPF, #42, 17-years).

#### 3.2.3. HIV prevention methods

Abstinence was recognised as a preventive strategy by both young women in participating and non-participating schools. One young woman noted, ‘*safe sex is about protecting yourself, your partner, as well as avoiding transmission of diseases, or actively abstaining from sex’* (NPF, #40, 16-years). Male participants, however, viewed abstinence as less practical: ‘*Abstaining is hardly encouraged among [us]’* (PM, #8, 19-years).

Condoms were widely mentioned by both young men and women as an important prevention method. Even though the use of condoms was commonly cited as a way to practice safe sex, they were primarily understood as a means to prevent pregnancy, especially among young women in both participating and non-participating schools. One young woman in the non-participating schools explained, ‘*in my case, I believe that we are worried about risks of getting infected with HIV, but also STIs. However, pregnancy is more important’* (NPF, #39, 17-years), while another noted, ‘*young people are more worried or preoccupied with pregnancy than with STIs and HIV’* (PF, #22, 18-years). Similarly, a young woman remarked, ‘*condoms allow us to protect ourselves’* (NPF, #41, 19-years) – though the context suggested a primary concern with avoiding unintended pregnancy. On the other hand, young men in participating schools were more likely to mention the dual protection benefit of condoms. One young man stated, ‘*the youth think that condoms prevent disease and pregnancy’* (NPM, #33, 16-years), reflecting an understanding of both HIV/STI and pregnancy prevention.

#### 3.2.4. Gender roles in safe sex decision-making

Perceptions of responsibility in practicing safe sex varied by programme exposure and gender. Among youth in participating schools, both young men and women emphasised shared responsibility. One young woman stated, ‘*responsibility for protecting oneself from risk during sex lies with both partners’* (PF, #15, 16-years), reflecting a more equitable and mutual understanding. This view was supported by a young man who noted, ‘*safe sex means that both partners know their HIV status’* (PM, #11, 16-years). In contrast, youth in the non-participating schools were more likely to express gendered views of responsibility. A young man noted, ‘*men are responsible for protecting themselves during sex’* (NPM, #32, 16-years), while a young woman said, ‘*the girl is the one responsible when it comes to sexual intercourse … this is why it is important to do the test’* (NPF, #45, 15-years). Another young woman added, ‘*the girl should be the one to refuse if she does not feel ready’* (NPF, #47, 15-years). These responses suggest among youth in non-participating schools; responsibility is not only individualised but also shaped by prevailing gender norms that burden young women with the moral and physical obligation to ensure protection. Conversely, youth in participating schools appeared to adopt more balanced views, pointing to a potential impact of the intervention in shifting gendered dynamics around sexual health decision-making.

## 4. Discussion

Our qualitative findings provide nuanced insight into how cultural norms, gender dynamics, and peer education exposure shape AYA dating behaviours and HIV perceptions in N’Djamena, Chad. A central theme was the motivation for dating, which appeared to differ by gender. Young women commonly emphasised emotional connection and companionship as their primary reason for dating, whereas many young men often described dating in terms of sexual fulfilment and demonstrating maturity. These patterns align with evidence from other sub-Saharan African contexts, which reveals distinct gender differences in AYA’s motivations for dating and sexual relationships. For example, (Ampofo, 1998) found that young women in Ghana prioritised love, affection, and the prospect of children in relationships – an intrinsic motivation – while young men’s narratives frequently included sexual conquest and multiple partnerships as markers of status (Meekers & Calvès, 1997). Such gendered patterns echo Buss’ sexual strategies theory, which suggests that women tend to invest in long-term, emotionally bonded partnerships while men pursue short-term mating opportunities to maximise sexual access (Buss & Schmitt, 1993). Locally grounded analyses further highlight the role of masculinity norms and peer dynamics in shaping these behaviours. In many African urban contexts, sexual prowess is closely tied to masculine identity, with young men often praised for having multiple partners as a sign of manhood. Qualitative studies report that this is perceived as a way to gain respect and ‘fame’ among peers (Khumalo et al., 2020), a behaviour believed to elevate their social status in male peer groups (Ezelote et al., 2024; Manyaapelo et al., 2019). This interplay of gendered motivations and social norms underscores the need to interpret AYA relationship behaviours in light of both universal theories and contextually specific frameworks in sub-Saharan Africa.

Partner selection criteria among Chadian AYA reflected a clear interplay between intrinsic and extrinsic factors. Many participants of both genders described intrinsic qualities such as a partner’s personality, values, or the desire to build a family as important in choosing whom to date (Khumalo et al., 2020). At the same time, extrinsic considerations like physical attractiveness and financial stability were also frequently mentioned, especially when discussing longer-term or material benefits of a relationship (Khumalo et al., 2020). Our findings mirror patterns reported in various African contexts. A study in Ghana by (Ampofo, 1998) among young women showed that love, affection, and the desire for children were crucial in partner selection, aligning with the intrinsic factors we identified. However, financial capability was also a significant consideration, resonating with our observations of extrinsic motivations (Ampofo, 1998). Similarly, research in Cameroon by (Meekers & Calvès, 1997)’s study noted that financial support can be a decisive factor for women, particularly in low-income contexts, as a relationship may provide economic survival or opportunity. In South Africa, many young women have been found to choose older male partners in part for financial benefits (Manyaapelo et al., 2019). These parallels suggest that while Chadian AYA value emotional connection, their choices are also shaped by the broader socioeconomic realities, a combination of deep-seated personal values and external incentives that is common across different African cultural settings.

Another important finding was the disparity in HIV knowledge and misconceptions between AYA who had received the peer education programme and those who had not. AYA in participating schools generally demonstrated a stronger grasp of HIV transmission routes and prevention methods, whereas those in non-participating schools, particularly young men displayed gaps and misunderstandings in their knowledge. Recent studies confirm significant disparities in HIV knowledge between AYA who participate in peer education programmes and those who do not (Ezelote et al., 2024). AYA lacking access to such programmes often retain dangerous misconceptions. For instance, in a Nigerian school-based intervention, the proportion of students with comprehensive understanding of HIV transmission and prevention jumped from 27% to over 80% after a peer-led education programme, whereas knowledge levels in a control group remained largely low (Ezelote et al., 2024). Even when aware of HIV risk factors, AYA often frame these behaviours as problems of ‘others’ rather than themselves. In our study, AYA readily identified high-risk behaviours such as alcohol use in sexual situations, multiple concurrent partnerships, and inconsistent condom use, but spoke of them chiefly as peer behaviours. For example, young men described a peer culture of competing over who would acquire the most sexual partners with little consideration for condom use, while young women frequently blamed friends for pressuring them into sexual relationships. This externalisation of risk-taking and youth often perceive danger more in their friends’ actions than in their own (Owino et al., 2024). Such perceptions indicate that even those educated about HIV remain aware of – and influenced by – high-risk social behaviours around them. This echoed (Swahn et al., 2021)’s findings on the overlooked roles of alcohol consumption and gender-based violence in HIV risk. Alcohol use further exacerbates these vulnerabilities as it emboldens young men to initiate condomless sex and leaves young women vulnerable to coercion, undermining their negotiation power (Duby et al., 2021; Owino et al., 2024; Plichta et al., 1991; Swahn et al., 2021). A recent South African study showed that frequent binge drinking significantly lowered the odds of condom use among young women, underlining how substance use, and gendered powered dynamics jointly heighten HIV risk (Singer et al., 2023). Ultimately, the disconnect between HIV awareness and protective behaviour among AYA persists, as social pressures and limited agency hinder the translation of knowledge into practice (Owino et al., 2024). This trend mirrors patterns observed across sub-Saharan Africa, where simply providing information is insufficient unless accompanied by efforts to shift peer norms and empower AYA to act on prevention knowledge (Chory et al., 2023). Peer-led and school-based programmes remain important to addressing these gaps; indeed, evidence shows such interventions can boost HIV knowledge and even uptake of testing services (Ezelote et al., 2024). However, experts emphasise that educational initiatives must also incorporate gender-transformative components, confronting harmful norms and building AYA’s confidence and skills in order to translate awareness into sustained protective behaviour (Singer et al., 2023; UNAIDS, 2024). Scaling up comprehensive, AYA-friendly interventions that engage both young men and women is essential to ensure no segment of AYA is left uninformed or unable to protect themselves (Bedingar et al., 2025).

HIV prevention in Chad has largely emphasised abstinence over comprehensive education (Bedingar et al., 2024). However, this approach fails to reflect AYA’s lived realities, especially in urban centres like N’Djamena, where later marriage ages give many young people greater opportunity to form premarital relationships from their mid-teens. In such a context, a strict ‘no sex until marriage’ message can ring hollow and instead foster stigma around premarital sexual activity, discouraging open conversation and education (Lo et al., 2016; Santelli et al., 2017). Indeed, evidence from sub-Saharan Africa shows that abstinence-only interventions have not effectively reduced HIV risk behaviours among AYA (Lo et al., 2016; Santelli et al., 2017), underscoring the limitations of this strategy. Addressing this disconnect requires a shift in societal norms and prevention messaging to align with AYA’s reality. Involving AYA and their families, educators, and community leaders is important for reshaping HIV prevention strategies in a more realistic and supportive way (Bedingar et al., 2025). Such broad engagement can help replace silence and shame with informed dialogue, breaking the taboos that hinder effective education. Peer-led health education initiatives also show promise for reaching young people on their own terms. For example, a recent Nigerian school-based programme significantly improved students’ HIV knowledge through peer educators (Ezelote et al., 2024). However, these interventions must be carefully designed with cultural nuance and community input to truly address the complex factors influencing AYA sexual behaviour and to avoid reinforcing stigma.

### 4.1. Policy and practice implications

This study suggests the need for comprehensive, gender-sensitive, and contextually grounded approaches to AYA sexual health education and HIV prevention in Chad. While our findings are not intended to be generalised, they point to important patterns and potential areas for programme improvement. Programmes may benefit moving beyond abstinence-only messaging to more accurately reflect the lived experiences of AYA, including the prevalence of premarital sexual activity and the influence of peer norms and gender power dynamics. Peer-led and school-based interventions appear promising in addressing knowledge gaps, particularly among young men and those outside formal education systems and could be considered for adaptation and scale-up. Such efforts might also incorporate gender-transformative content that encourages young women’s agency and engages young men in rethinking harmful masculinity norms. Community-level engagement, including parents, educators, and religious leaders, may help to shift stigmatising attitudes and foster more open dialogue around AYA sexuality. Finally, expanding access to AYA-friendly services and accurate information on all available prevention methods, including dual protection strategies with condoms, remains an important priority. These exploratory insights can inform the design of more responsive and inclusive interventions. A summary of potential policy and practice considerations is included in S2 Appendix.

### 4.2. Limitation

This study included some limitations. Firstly, the study utilised descriptive phenomenology, a method that can potentially lead to researcher bias influencing everything from the research question’s development to data interpretation. To mitigate this, efforts were made to reduce the impact of the researchers’ personal beliefs, values, and experiences. This was achieved by thoroughly reviewing and discussing focus group discussion (FGD) transcripts before conducting interviews, thereby ensuring that any assumptions made by the interviewer did not overshadow the participants’ actual experiences. Secondly, our study focused solely on high school students, thereby excluding AYA not enrolled in formal education, who represent approximately 70–82% of the Chadian AYA population. This limits the generalizability of the findings to out-of-school youth who may face different vulnerabilities and access barriers to HIV-related information and services. Thirdly, the study did not include AYA from key populations most at risk for HIV, such as AYA involved in sex work, MSM, transgender individuals, and injection drug users. Given their heightened exposure to HIV risk, the absence of these groups presents a significant gap. Finally, thematic saturation was not reached. However, this aligns with the explanatory nature of the study, which aimed to generate preliminary insights rather than provide exhaustive thematic coverage. The sample was designed to reflect a diversity of perspectives rather than to capture all possible variations. We have been transparent in presenting emergent themes and acknowledge that future studies with larger and more targeted samples are needed to further explore and deepen understanding of the identified issues.

## 5. Conclusions

The study provides a comprehensive look at the complex interplay between adolescent dating behaviours and HIV perceptions among AYA in urban Chad. It reveals clear gender-based differences in dating motivations and partner selection, as well as disparities in HIV knowledge and risk awareness, particularly between AYA exposed to peer education programmes and those who were not. These findings underscore the urgent need for targeted, AYA-friendly, and gender-sensitive interventions that go beyond abstinence-focused messaging to reflect the lived experiences of AYA. Moving forward, research and programming should prioritise the lived experiences of AYA and actively engage community stakeholders such as educators, parents, and religious leaders in promoting open, non-stigmatising dialogue and comprehensive sexual health education. Aligning prevention strategies with the social and behavioural contexts of Chadian AYA is essential to equip them with the knowledge, skills, and agency to make informed decisions and reduce their vulnerability to HIV.

## Supplementary Material

S1

S2

S3

Supplemental data for this article can be accessed online at https://doi.org/10.1080/17441692.2025.2534619.

## Figures and Tables

**Table 1.** Sociodemographic characteristics of participants.

Participant characteristics	Participating schools (%)	Non-participating schools (%)	Total N (%)

High school student sex			
Male	12 (25%)	12 (25%)	24 (50%)
Female	12 (25%)	12 (25%)	24 (50%)
High school grade			
9^th^ grade	0 (0%)	6 (100%)	6 (12.5%)
10th grade	8 (47%)	9 (53%)	17 (35.5%)
11th grade	11 (61%)	7 (39%)	18 (37.5%)
12th grade	4 (57%)	3 (43%)	7 (14.5%)
Mean age (years) [Mean (SD)]			
All groups	16.75 (0.90)	–	–
Males in participating schools (PM)	16.92 (0.90)	–	–
Females in participating schools (PF)	17.25 (0.97)	–	
Males in non-participating schools (NPM)	–	16.58 (0.90)	–
Females in non-participating schools (NPF)	–	16.25 (1.14)	–

**Table 2.** Emergent thematic levels.

Themes	Sub-themes	Codes

Culture of dating	Reasons for dating	Seeking companionship; Sharing ideas; Good company; Peer pressure; Curiosity; Need satisfaction; Pleasure
	Partner selection criteria	Emotional attraction; Family-oriented goals; Physical attraction; Financial rewards; Non-financial rewards
Awareness and risk-taking behaviours	HIV transmission	Mother-to-Child Transmission; Unprotected sex; Soiled objects; Blood transmission; Blood pacts
		Weight loss; itching; and diarrhea
	HIV risk-taking behaviours and prevention methods	Abstinence; Sexual vagrancy; Safe sex; PrEP; Condoms; Practicing secondary abstinence; Dual protection nature
	Gender roles in safe sex practice decision-making	Shared vs. individual responsibility; Prevention; HIV testing

**Table 3.** Selected quotes for the dating culture among AYA in Chad.

Themes	Subthemes	Codes	Selected quotes

Culture of dating	Reasons for dating	Seeking companionship	‘A friend to share ideas with’ (PM, #4, 19-years)
		Peer pressure	‘I believe what motivates young people like me to practice sex is peer-pressure and desire’ (PF, #20, 15-years)
		Motivations driven by sexual behaviour	‘Need satisfaction’ (NPM, #25, 16-years)
			‘Men of our age have sex just to satisfy themselves and for pleasure’ (NPM, #28, 17-years)
	Partner selection criteria	Intrinsic motivation	‘The culture around dating is focused on emotional attraction and sharing ideas’ (PF, #21, 18-years)
			‘... end up in a marriage around the family and having common projects’ (PM, #5, 16-years)
		Extrinsic motivation	‘Our choice of partners is based on physical appearance’ (PM, #3, 17-years)
			‘We date men because of money’ (NPF, #39, 17-years)
			‘For me, I choose my partner based on the fact that she is helpful, and she offers me gifts’ (NPM, #26, 16-years)

## Data Availability

The data underlying this article cannot be shared publicly due to ethical restrictions. When applying for ethical approval, we did not specify that the data would be made publicly available in a repository. As part of the written and verbal consent, we assured participants that all data would be confidential and access to the recordings would be restricted to the research team. We did specify that ‘some of their words’ may be used in reporting the findings of the study (which we have done within the manuscript as non-identifiable quotes), however, to make all raw data publicly available will be a serious breach to the rights of ethical of participants given did not consent to this.
