# Supplementary material for: Adolescents and young adults dating and HIV perceptions: A phenomenological study in N’Djamena, Chad
Source: Glob Public Health. Author manuscript; Available in PMC 2025 Dec 1. (PMC12667022; doi:10.1080/17441692.2025.2534619)
Supplement: S1 [file NIHMS2115640-supplement-S1.docx]

**Supporting Information File 1**

**Brief Background:**

From 2017 to 2021, IBC has implemented the life skills program in classrooms in N’Djamena to increase knowledge and skills needed for healthy relationships, effective communication and responsible decision-making when it comes to HIV related risk, this intervention consisted in teaching life skills lessons through peer education in a holistic approach. This study aims to ascertain coverage, perceptions and beliefs of the youth on HIV and AIDS, as well as understand the influences that may impact intentions to practice safer sex.

The study participants are the youth (aged 15-24) in both participating and non-participating high schools located in N’Djamena. These participants were selected randomly from the purposive list of participating and non-participating high schools in N’Djamena.

**Topic Guide**

Hello, my name is…I am working with IBC on research that aims to learn more about HIV knowledge and self-efficacy among the youth in high schools in N’Djamena. I will be conducting a focus group discussion, which will last 90-120 minutes. Please refer to the consent form for more details.

Consent Process

1. Please introduce yourselves
   1. Please share with us information about your high school and class?
2. What is the culture around dating?
   1. Probe: At what age do young people start dating?
   2. Probe: How do young people select their partners?
   3. Probe: What does dating involve?
3. How do young people talk about sex with their friends?
   1. Probe: Do young women/men of your age talk about sex with friends?
   2. Probe: How do women/men of your age talk about it?
4. Whom or what do young people rely on for information?

*School sex education (where provided)*

- 1. Probe: How do you feel about the sex education that is provided in school?
  2. Probe: To what extent has your knowledge changed as a result of the IBC project?
  3. Probe: How could it be improved upon?

*School sex education (where not provided)*

- 1. Probe: How do you feel about school teaching young people like yourselves about relationships, sex and contraception?
  2. Probe: Do you think young people would find the introduction of classes on sexual issues useful?

1. What do you know about HIV/AIDS?
   1. Probe: What is HIV/AIDS?
   2. Probe: How is HIV/AIDS transmitted?
   3. Probe: What are the symptoms of this disease?
2. How do young people of your age usually find out about relationships, sex and contraception?
   1. Probe: Who shaped your beliefs on HIV/AIDS?
   2. Probe: Whom or what do young people rely on for information?
3. What are risky behaviors related to HIV/AIDS? If person does not know what risk behavior is, explain that is mainly about sexual activities without a condom).
   1. Probe: To what extent do you think that people of your age take risks of any sort during sex?
   2. Probe: Are young people more worried or concerned about pregnancy or HIV/AIDS or other STIs?
4. How can you do to prevent HIV/AIDS?

*Risk prevention*

- 1. Probe: What are the ways in which you can prevent HIV/AIDS transmission?
  2. Probe: Who should be responsible for protecting against any risk during sex?
  3. Probe: What does safe sex mean to young people?
  4. Probe: How do young people feel talking about contraception with partners?

*Condoms*

- 1. Probe: What do young people think about condoms?
  2. Probe: Where do young men and women generally obtain their condoms from?
  3. Probe: What do you think would make people of your age adopt “safe sex” practices?

*PrEP*

- 1. Probe: What do you know about PrEP?

Probe: In what ways have young people been getting hold of PrEP?

- 1. Probe: Are there other ways that people are obtaining PrEP?
  2. Probe: How did you find information about PrEP?

*Abstinence*

1. Probe: Do young people of your age are actively abstaining from having sex?
   1. Probe: Is abstinence actively promoted?

IF NOT, ask questions around reasons for having sex:

- - 1. Probe: Why do you think women/men of your age have sex?
       1. Probe: What do you think they get out of it?
       2. Probe: What do you think it means to them?

1. How do young men/women usually find out about services (health centres, young clubs, organizations)?
   1. Probe: Can you list for me all the places and people young people are able to talk to find out about sex, contraception, STIs?
   2. Probe: Do young men and women of your age visit the local services for contraception and sexual health advice?
      1. IF YES,
         1. Probe: Why do young men/women usually attend services?
         2. Probe: Is there anything that would stop young people from going?
2. What do you think are the most important features of a sexual health service for young people?
   1. Probe: Are there differences in the needs of young men and women?
   2. Probe: Where do you think people’s sexual health services should be held (location)?
   3. Probe: Who should provide the information and advice?
   4. Probe: How do you think the services in your locality could be improved upon?
   5. Probe: What do you think are the best ways of advertising and promoting services?
   6. Probe: Can you think of 3 words which are the most important to use when advertising and promoting sexual health services for young people?

Do you have anything else you’d like to share with us! Thank you for your time!
