# Supplementary material for: Adolescents and young adults dating and HIV perceptions: A phenomenological study in N’Djamena, Chad
Source: Glob Public Health. Author manuscript; Available in PMC 2025 Dec 1. (PMC12667022; doi:10.1080/17441692.2025.2534619)
Supplement: S2 [file NIHMS2115640-supplement-S2.docx]

**Appendix 2. Policy and practice implications for AYA HIV prevention in Chad**

| **Key findings** | **Recommendations for policy and practice implication** | **Responsible stakeholders** |
| --- | --- | --- |
| ***Gender motivations and social norms drive AYA relationship dynamics*** | | |
| Young men are expected to assert sexual prowess, while young women often lack power to insist on protection. | 1. Design HIV prevention programs that incorporate gender-transformative approaches; 2. Challenge harmful masculinity norms and promote respectful, equitable relationships; 3. Empower young women with negotiation skills and engage young men as allies in promoting consent and shared responsibility for protection. | 1. Ministry of Health; 2. Ministry of Education; 3. Youth and gender-focused NGOs or programs; 4. School administrators and teachers; 5. Community and religious leaders. |
| ***Peer dynamics fuel risk-taking*** | | |
| AYA face strong peer pressure to engage in risky behaviors, including drinking alcohol before sex, having multiple partners to “fit in”. These social norms can normalize unsafe practices. | 1. Leverage peer influence for good; 2. Implement peer-led interventions and school clubs that promote positive role models and healthy behaviors; 3. Incorporate life-skills training such as communication, refusal skills into curricula so AYA can resist negative peer pressure; 4. Engage population AYA influencers to spread safe-sex messages that counter the idea that risk-taking is cool. | 1. Schools, including teachers, counselors establishing peer clubs; 2. AYA organizations and NGOs running peer educator programs; 3. Community AYA leaders and mentors; 4. Parents supporting AYA participation in healthy group activities. |
| ***Uneven access to HIV knowledge and life-skills education*** | | |
| AYA who received school-based peer education program showed better HIV knowledge and more egalitarian attitudes, whereas many uninvolved AYA, especially young men remain misinformed and retain traditional views. | 1. Scale up comprehensive sex education coverage; 2. Expand peer-education and life-skills programs to reach all schools and out-of-schools AYA, ensuring accurate HIV information for everyone; 3. Institutionalize these programs into the national curriculum so they are sustained; 4. Tailor content to address young men’s misconceptions and encourage gender-equitable attitudes among all students. | 1. Ministry of Education and National AIDS Program implementing nationwide curriculum reforms; 2. NGOs and agencies training peer educators and facilitators; 3. School authorities allocating time and resources for life-skills sessions; 4. Donors and partners funding scale-up to additional schools and communities. |
| ***Overemphasis on abstinence and stigma around AYA sexuality*** | | |
| Current sex education often stresses “no sex before marriage” to avoid pregnancy, but this disregards the reality of premarital sexual activity and deters open discussion. AYA are more worried about pregnancy than HIV, and abstinence-only messaging can stigmatize sexually active AYA, leaving them without support or accurate information. | 1. Adopt a comprehensive sexuality education (CSE) approach that balances messages about abstinence with realistic education on contraception, HIV/STI prevention, and healthy sexual decision-making; 2. Update curricula to address premarital sex in a non-judgmental, fact-based manner, emphasizing condom use for dual protection and the importance of testing and mutual monogamy for those who are sexually active. 3. Promote AYA-friendly SRH services that prioritize confidentiality, accessibility, and non-judgmental care. 4. Conduct community sensitization with parents and religious leaders to reduce stigma and gain buy-in for CSE. | 1. Ministry of Education; 2. Teacher training institutes; 3. Media organizations; 4. Health educators and counselors (health providers; community health workers); 5. Parent associations; 6. Community and faith leaders. |
